# The limited application of stem cells in medicine: a review

**DOI:** 10.1186/s13287-017-0735-7

**Published:** 2018-01-02

**Authors:** Jordan Poulos

**Affiliations:** University College London Medical School, London, UK

**Keywords:** iPSC, hESC, Stem cell ethics, Stem cell regulation, Stem cell politics, STAP, Durisotto vs Italy, X-Cell Centre, MSC, DCM

## Abstract

The history of stem cell therapies is one of a limited number of clinical applications despite a vast therapeutic potential. Major breakthroughs in stem cell research have not yet enjoyed clinical success—all stem cell therapies bar hematopoietic stem cell transplantations remain experimental. With the increased risk of organ failure and neurodegenerative disease associated with our ability to push the boundaries of life expectancy comes an increased pressure to pioneer novel stem cell-based therapeutic approaches. We conclude that the failure of such therapies to achieve clinical translation stems from the polarising effect of the ethical debate around their use. The intractability of the ethical debate is double edged: legislators not only have placed tighter restrictions on certain stem cell therapies, but do so in favour of less controversial cells which will have worse outcomes for patients. It is by considering this relationship between the politics, ethics and science of stem cells that the reasons for the currently limited clinical significance of stem cell therapies be realised.

**Electronic supplementary material:**

The online version of this article (10.1186/s13287-017-0735-7) contains supplementary material, which is available to authorized users.

## Background

The following paper is a review of the current role of stem cells in medicine in the context of political, ethical and scientific limitations, which draws on the findings of like-minded reviews and research papers. The research question this paper seeks to address is a simple one: why has such a promising area of regenerative medicine as of present yielded such a limited impact?

Through a comprehensive review of the state of the ethical debate, regulation and the process of clinical translation, this paper has reached the conclusion that it is a combination of these three factors, alongside the outcome of the ongoing debate as to whether to prioritise research using somatic-based therapies over human embryonic stem cells (hESCs), which has limited the role of stem cells in medicine.

**Fig. 1:**
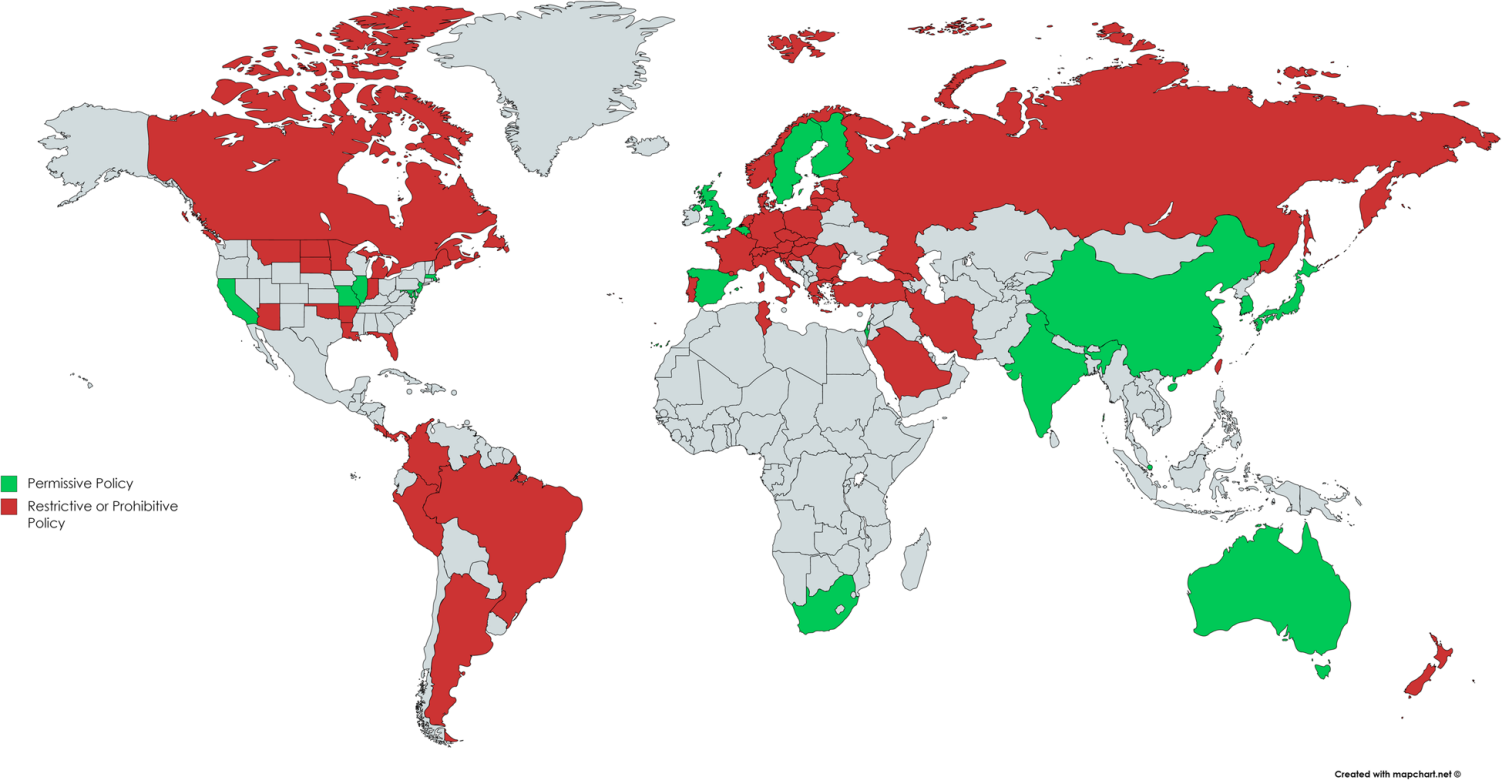
Restrictive and prohibitive stem cell policy worldwide. Permissive policy was defined as a policy which specifically permits somatic cell nuclear transfer (SCNT) under certain conditions. Restrictive policy was defined as one which prohibits SCNT, permits the use of hESC research using supernumerary in vitro fertilisation embryos or only permits hESC research on a limited number of lines. Prohibitive policy was defined as one in which research on hESCs or their products was prohibited. Given the differences in policy between federal states in the US, this country was omitted from the calculation of the percentage of countries with restrictive or prohibitive hESC policy. Based on data from the Hinxton Group

The majority of this paper relies on secondary data but does include examples of primary data, such as Fig. 1, which shows restrictive and prohibitive stem cell policy worldwide, and Table 1 in Appendix 1, compiled using data from a freedom of information request to the Department of Health regarding stem cell funding. (I can confirm that I have permission to use any figures that are not original.)

## Political regulation

Through setting the parameters which define the scope of stem cell-based therapies in medicine, regulation can be seen to be a reflection of the state of the ethical discourse surrounding stem cells. This process can be seen in the decision by the House of Lords to prioritise adult stem cell research over embryonic stem cell research with an emphasis that both be considered for therapeutic applications [1]. The intrinsic relationship between stem cell politics and stem cell ethics can be traced back to the Warnock Report [2], which advised giving the human embryo legal protection through a “special status” whereby embryonic research can only take place if there is no viable alternative. By and large, this special status remains respected in stem cell regulation, such as the requirement for an embryo research oversight (EMRO) process to assess the ethical justification for all research involving the preimplantation stages of human development under the International Society for Stem Cell Research (ISSCR) guidelines [3] and the prohibition of Horizon 2020 EU funding for research which creates hESCs solely to procure stem cell lines [4]. As such, the pressure from ethical opponents to hESCs to show somatic stem cells have therapeutic value equal to or greater than that of hESCs influences their regulation. Of the countries with specific legislation in place regarding hESC research, 77% are either restrictive or prohibitive [5] (Fig. 1). It should be noted, however, that hESC regulation in the UK strikes the right balance between creating enough space for scientific research and respecting the moral convictions of those opposed to hESC research.

### Regulatory guidelines and legislation

Advances in both stem cell technologies and cloning following the turn of the century, such as the isolation of highly multipotent mesenchymal stem cells (MSCs) from umbilical cord tissue and amniotic fluid [6], and the reprogramming of somatic cells into induced pluripotent stem cells (iPSCs) [7], created pressure on the UK government to amend the 1990 Human and Fertilisation Embryology Act (HFEA) [8]. The original HFEA had significantly liberalised Britain’s embryonic research regime [9] through the legalisation of licensed research on intact embryos in vitro during the first 14 days following fertilisation and prior to the appearance of a primitive streak [8]. The primitive streak is the point at which the blastocyst (inner cell mass) of the embryo differentiates into the three germ layers which give rise to adult tissue: ectoderm, mesoderm and endoderm. By allowing for embryonic research to take place within a limited timeframe, the 14 day rule sought to reconcile the regenerative benefits of embryonic research with the need to protect the “special status” of the unborn [10].

The ISSCR’s policy on the in vitro research timeframe for human embryos is consistent with that of the HFEA: prohibition of the in vitro culture of preimplantation embryos beyond 14 days or after the appearance of the primitive streak [3]. Although the 14-day rule represents a viable political compromise between enabling scientific inquiry and accommodating for diverse moral concerns in human embryo research, it is becoming an increasingly arbitrary line in the sand. The ability to aggregate synthetic human entities with embryo-like features (SHEEFs) offers a way of synthetically replicating embryonic development [11]. Since SHEEFS are both synthetic and non-intact embryos, they fall outside of the remit of research limits placed by the HFEA. Two recent studies have also reported the culturing of autonomously growing human embryos in vitro up to the 14-day mark [12, 13] (previously culturing of human embryos had not been reported beyond 9 days [14]). Both studies had to discard the human embryos on the 14^th^ day in compliance with existing legislation. If the 14-day rule was extended, disorders of pregnancy such as miscarriage and developmental abnormalities could be fully investigated. The failure to readjust this line in light of recent scientific advances risks sacrificing embryonic research during later embryogenesis, and any resulting therapeutic benefit from it.

Somatic cell therapies, including iPSCs and MSCs, are regulated as advanced therapy medicinal products (ATMPs). All ATMPs are subject to a centralised marketing authorisation procedure involving a 210-day assessment for quality, safety and efficacy by the CAT (Committee for Advanced Therapies). EU regulation allows member states to authorise hospitals on a national scale to use ATMPs without marketing authorisation, known as the “hospital exemption” clause [15]. The basis of such an exemption is to allow non-commercial ATMPS with enough evidence for therapeutic use to be received by an individual patient under the exclusive responsibility of a medical practitioner. A public consultation into ATMP regulation in Europe deemed the high requirements of the regulation as responsible for the disappearance of innovative products as well as discouraging to new developments [16]. Contributors also viewed this as preventing the majority of ATMPs being used beyond a role under the “hospital exemption” clause. For example, the data requirements as part of CAT authorisation fail to distinguish between the disease, target patient and type of product (including whether the product is autogenic or allogenic). Cytori, a stem cell research company, recently withdrew from the European market due to regulatory hurdles facing autologous therapies [17]. It is difficult for ATMP regulation to allow for the rollout of a larger quantity of therapies over a shorter timescale whilst protecting a high standard of safety and efficacy. Although further convoluted by an ethical minefield, the exact same challenge faces embryonic stem cell regulation.

### The political response to unproven therapies

Unproven stem cell therapies increase the risk of therapeutic misestimation (where patients incorrectly estimate the probability of benefit or risk [18]), jeopardises the reputation of legitimate therapies which are yet to be commercialised and fans misconceptions regarding the current state of scientific and clinical developments. Whilst a political consensus exists around tighter regulation, the market for unproven therapies appears to be expanding. In Australia, the number of private stem cell clinics has exponentially increased from two to over 40 since 2011 [19], under a medical practice exemption clause which enables autologous stem cell therapies for individual patients to be outside of the remit of the Therapeutic Goods Administration (TGA) [20]. Similar exemptions in the EU include “compassionate use” programmes—in which a patient with life-threatening, long-lasting or debilitating illness who cannot be treated by an authorised medical product accesses an investigational drug outside of a clinical trial [21]. It was the issue of compassionate use which underpinned the *Durisotto vs Italy* [22] case concerning Stamina therapy in the European Court of Human Rights.

Under a compassionate use framework, a private group of clinicians offered Stamina therapy, an unproven therapy involving the neural differentiation of allogenic MSCs to treat neurological disorders, to patients in northern Italy in 2011 through the Stamina Foundation [23]. The only evidence supporting the method was in self-reports provided by treated patients [24]. Although Italian law allows for the compassionate use of cell therapies, the therapy must be scientifically justifiable, informed consent must be exercised by patients and approval must be given by an ethics committee [25]. An investigation by the Italian Medicines Agency, Agenzia Italiana del Farmaco (AIFA), found that none of these conditions had been met, and issued an inhibitory order to shut down the activities of the Stamina Foundation. Under pressure from pro-Stamina activists, the Italian government overturned the AIFA decision under a decree by the Ministry of Health in March 2013, allowing the Stamina Foundation to continue to treat existing patients. In addition, the House of Representatives provided €3 million for a public clinical trial to test the safety and efficacy of the Stamina method [23]. By doing so, the Italian government had defined the regulatory requirements designed to protect the safety of patients as red tape barring access to therapeutic benefit. Such a decision in turn created a heterogeneous approach to the translation of unproven stem cell therapies across the EU, resulting in legal loopholes which can be exploited by providers to generate a market for European stem cell tourism.

The *Durisotto vs Italy* case was an appeal to the European Court of Justice to obtain stamina therapy on compassionate use grounds for new, rather than existing, patients under the 2013 Italian government decree. The appeal was framed in terms of the violation of several articles of the European Convention of Human Rights, namely Article 2 (right to life), Articles 8 (right to respect for private life) and 14 (prohibition of discrimination) [22]. Hence, *Durisotto vs Italy* sought to determine whether the principles of the European Convention of Human Rights could justify the compassionate use of an unproven therapy [24]. The European Court of Justice deemed that Durisotto’s claim for compassionate use was inadmissible. This was drawn on the basis that, in the absence of substantiating scientific evidence, the March 2013 decree “pursued the legitimate aim of protecting health” [22]. *Durisotto vs Italy* not only demonstrated how human rights can be politicised as a ploy to bypass legislative measures designed to protect safety around unproven stem cell therapies, but how a counter argument exists to “the right to life” in gaining compassionate use: there exists a “right to protect life”.

The importance of defining the safety and efficacy of unproven stem cell treatments before they are marketed to the public can be seen in the case of the X-Cell Centre, a private stem cell clinic operating in Dusseldorf providing unproven transplantations of autologous bone marrow stem cells for neurological disorders. Even after the severe internal bleeding in the head of a 10-year-old boy following cell injections in the brain, and the death of an 18-month-old child after a similar procedure [26], the centre remained open through a legal loophole. German law allows for an 18-month transition period following meeting new EU legislation after its implementation [27]. This enabled the X-Cell Centre to continue to operate in the absence of experimental licences after regulation which enforced licence application for experimental therapies was implemented in German law in 2009.

There are clear political lessons to be learnt from the X-Cell Centre fiasco. Firstly, since the market for stem cell tourism largely rests on therapeutic misestimation, there should be a greater effort from the medical and scientific communities to increase public awareness regarding the clinical safety of stem cell treatments. Indeed, the generality of the term “stem cell”, combined with the commercial availability of unapproved therapies, often means that patients cannot distinguish between experimental interventions and proven ones [28]. Secondly, regulatory loopholes, such as the one which allowed the X-Cell Centre to continue to operate whilst compromising patient safety, should be closed. Thirdly, EU legislation should allow for private stem cell clinics to be shut down following an investigation into serious adverse events. Far from being the norm, therapeutic benefit from experimental therapies is the exception [24]. By failing to keep that in mind, the political response to unproven therapies is incoherent. For as long as it is, patients will be endangered, and our expectations of stem cell therapies distorted.

## The ethical implications of stem cell therapies

The derivation of the first hESC line in 1998 [29] ignited scientific interest in hESCs as a regenerative tool, as well as creating one of the most heated and intractable debates in medical ethics. In the absence of transparency over funding for stem cell research (Appendix 1), the terms of this debate have been limited. The inability to define the start point of personhood has rendered the argument of opponents to hESCs as unfalsifiable; no means exist to prove or disprove the idea that personhood begins at conception. As a result, scientific evidence will always be secondary to the assumption that there exists a moral status in the human embryo. Conceding this moral status and framing it in terms of its potential therapeutic gain is a flawed strategy. If the moral status of the embryo is conceded, then this raises the issue of why it should not be protected in the first place. Such an ethical impasse has, in turn, driven stem cell science in the pursuit of less ethically fraught cells with similar therapeutic value as hESCs. Yet, such cells, namely iPSCs and MSCs, have failed to live up to therapeutic expectations. With no means of both allaying the ethical implications around the use of hESCs and imitating their therapeutic value, the impasse widens.

### The ethics of stem cell research and interventions

The words of John Danforth, in response to the now tabled bills in the Missouri Senate and House in 2005 which sought to introduce a state-wide ban of somatic cell nuclear transfer (SCNT) [30], encompasses the division between proponents and opponents of stem cell therapies. According to Danforth, the proposal to criminalise SCNT, a technique used to procure stem cells using an ovum and a donor nucleus from a somatic cell, “calls for a choice between two understandings of human life. On the one hand, we have the millions of people who suffer from ALS, Alzheimer’s, juvenile diabetes, Parkinson’s, spinal cord injuries, and cancer—and the loved ones who care for them and suffer by their sides. On the other hand, we have tiny bundles of unfertilized cells existing in Petri dishes. Supporters of the legislation should explain to the afflicted and their loved ones why they care more about those cell bundles than they do about the people” [31]. The issue is that arguments such as the one made by Danforth make a critical error in oversimplifying the ethical opposition to SCNT.

The thinking behind the failed Missouri bill was based on the principle that since SCNT involves the procurement of stem cells from the inner cell mass of the blastocyst removed from an electrofused egg cell which is subsequently discarded, any resulting therapeutic benefit from these cells is considered as unethical [32]. Arguments made by proponents of stem cell research, such as Danforth’s, give greater credence to the potential therapeutic benefit from stem cells derived via SCNT than the belief that discarding a fertilised egg is morally objectionable. The problem with such arguments is that they *assume* this hierarchy of moral thought is accepted. Our view of whether there exists a moral status in the embryo hinges on the point at which we define the onset of personhood. Since the start point of personhood is an unfalsifiable concept, there is arguably no right to attempt a trade-off between the moral standing of the unborn with its potential regenerative benefits.

Stern ethical opposition on the back of this argument has resulted in more successful anti-SCNT and restrictive hESC legislation, such as the prohibition of SCNT for research purposes in Australia [33] or the Bush administration’s policy to limit federal funding only to 21 existing hESC lines in 2001 [34]. The limited number of existing stem cell lines receiving funding under Bush’s policy was wholly inadequate in terms of its genetic diversity for the recipient population [35]. Without federal support, the development of additional lines could only take place via investment from nongovernment sources until the policy was revoked by the Obama administration in 2009 [36]. Even so, the Obama administration still sought a middle ground through the Omnibus Appropriations Act, which contained the Dickey-Wicker provision to prohibit federal funding for “the creation of embryos for research processes or research in which human embryos are destroyed, discarded or knowingly subjected to risk of injury” [37]. Obama’s policy had the effect of broadening access to existing hESC lines whilst maintaining the barrier to the creation of new lines, such as ones focusing on specific types of stem cell with new genetic characteristics. The Dickey-Weaver clause also spawned a policy crisis in the form of an injunction by US District Judge Royce Lambeth against federal funding for ESC research on the grounds that current ESC research guidelines violated the provision [38]. Before it could be lifted, the injunction had already impacted research grants worth $140 million [39]. These policy decisions, seen as a quick fix to complexity of the ethical impasse, have pushed the field of stem cell science into seeking alternative methods of generating less ethically divisive stem cells.

Although both iPSCs and MSCs have exciting therapeutic potential, there are still several reasons to be concerned that such cells are not yet fit for experimental purpose. MSCs can be easily isolated from fat, umbilical cord tissue and bone marrow and have not yet shown adverse effects during systemic administration, in part due to their immune modulatory effects [40]. They have been shown to produce an anti-inflammatory response in vivo for models of graft versus host disease (GvHD), inflammatory bowel disease, allergic airway disease and multiple sclerosis [41–44]. After migrating to the site of infection in response to inflammatory cytokines, MSCs can produce either a pro-inflammatory or anti-inflammatory response depending on the type of toll like receptor (TLR) displayed by immune cells [45–47]. Through the production of immunomodulatory soluble factors (interleukin-10, prostaglandin E2, indoleamine 2,3 dioxygenase, nitric oxide and TSG-6 [48–50]), the proliferation and function of major immune cells such as T lymphocytes, dendritic cells and natural killer cells can be suppressed. Due to their low immunogenicity and immune modulatory properties, allogenic grafts using MSCs are less likely to incur immune rejection and more likely to prolong skin graft survival [51]. Similarly, reprogramming the somatic cells of a patient would produce an autologous graft of iPSCs instead of the allogenic graft derived from hESCs, mitigating the risk of immune rejection associated with the use of hESCs in transplantation.

However, the indefinite growth capacity and plasticity (differentiation potential) in hESCs remains unrivalled, meaning that hESCs can be grown for a longer duration in culture. Although embryonic pluripotency is short lived, capturing it under in vitro conditions provides unlimited access to tissue for transplantation, of any cell type [52]. Unlike with hESCs, ageing has been shown to significantly reduce the survival and differentiation potential of MSCs derived from bone marrow [53]. MSCs have also been shown to be unable to differentiate uniformly, and embolise in the lungs when injected intravenously, causing epithelial damage [54, 55]. Recorded evidence of an iPSC’s “epigenetic memory” of its donor cell of origin [56] results in a skewed differentiation potential; alternative cell fates are restricted by the fact that an iPSC has a tendency to differentiate along lineages related to the donor cell. In addition, studies have shown iPSCs to differentiate less efficiently than hESCs. For instance, a comparison of the efficiency of the neural differentiation of hESCs and iPSCs found that 90% of ESCs differentiated into neuroepithelial cells, compared to 15% of iPSCs [57]. It should be noted, however, that certain lines of iPSCs have been shown to have a differentiation efficiency greater than or equal to that of hESCs, and vice versa [58]. The general consensus is that efficiency varies between cell lines, rather than the stem cell type.

Due to the integration of oncogenes in the reprogramming process which can potentially cause cancer by insertional mutagenesis [59] and have been shown to disrupt tumour-suppressing genes [60, 61], iPSCs have a significant tumorigenic potential. One of these oncogenes, c-Myc, when introduced by a retrovirus vector, resulted in tumour formation in 40% of mice chimeras by activating previously silenced genes [62]. To get around this problem, several techniques deriving iPSCs without transgenes, such as mRNA and protein transfections, have not yet reported tumour formation in chimeras [63]. However, it remains difficult to create a safe reprogramming approach that has both high efficiency and does not result in teratoma formation.

Focusing on the potential of iPSCs and MSCs alone as a means to sidestep discussion around the moral status of the human embryo has created a disparity between therapeutic expectations (based on the properties of stem cells) and therapeutic outcomes (based on safety and efficacy outcomes). Even for existing bona fide stem cell therapies, such as in hematopoietic stem cell transplantation (HSCT), clinical benefit is still limited by several factors including graft failure, GvHD, limited donor availability and morbidity. For as long as the disparity between therapeutic expectations and outcomes continues to exist, the application of stem cells in medicine will be perceived as limited.

## The scientific barriers to clinical translation

After meeting regulatory requirements, stem cell products pass through the clinical translation pathway. This process from bench to bedside acts as a filter against therapies with safety risks and low efficacy. As a result, the translational pathway has failed to produce stem cell therapies beyond their current role in treating blood and immune disorders such as sickle cell disease (SCD). The transplantation of stem cells into ectopic locations presents difficult to appraise risks such as tumour formation and post-transplant complications. In order to address this risk, the ISSCR guidelines for clinical translation allows for attempting stem cell-based interventions “only in exceptional circumstances” [3]. Striking a balance between clinical progress and scientific caution by leaving enough room for innovation, the ISSCR’s judgement acknowledges the fact that beyond the ethical and political discourse, stem cell therapies are not yet ready to be pioneered on patients.

### The viability of potential stem cell therapies

With ESC-based therapies sidelined by ethical and political opposition, the advancement of stem cell science lies in expanding the use of somatic stem cells beyond their current role in replacing cells of their own lineage. Attempts to do this can be seen in treatments for neurological disorders as well in toxicological applications (Appendix 2).

Despite the successful derivation of neural and glial cells from hESCs, MSCs and neural stem cells (NSCs), uncertainties around both the mechanism by which transplanted stem cells result in functional recovery and the right type of stem cell to use in cellular grafts must be addressed before stem cell-based neurological therapies are used in a clinical setting [64]. The effect of novel treatments as of yet cannot be defined beyond correlation with improved brain recovery. Due to this, our understanding of the mechanism of such treatments is limited. Considering not only the anatomical complexity of the brain, but also the inability to guarantee the controlled migration of transplanted cells, it is unlikely that neurological disorders will be the first beneficiaries of clinically approved stem cell therapies. Instead, the most viable therapies are treatments for conditions with cell autonomous defects and involve the loss of a single type of cell [65]. This is not to say that promising clinical trials to treat neurological disorders should not be widened to treat a greater number of patients. Rather, the continued documentation of the optimal trophic factors, cell dosage and cell implantation locations [66] which result in functional recovery will nudge stem cell-based therapies for neurodegenerative diseases further towards the goal of clinical translation.

The general trend in novel cell replacement strategies appears to be that producing cells in vitro is not the problem, but rather the safe and effective engraftment of transplanted cells in the host. This trend can be seen in attempts to treat liver cirrhosis from hepatocytes derived from iPSCs and the engraftment of cardiomyocytes to treat cardiac disease [65]. A similar challenge faces novel stem cell treatments for type 1 diabetes, an autoimmune disease which causes beta cells of the islets of Langerhans of the pancreas to be destroyed. Type 1 diabetes can be treated by the transplantation of cadaveric pancreata and islets to temporarily restore glycaemic control before the patient relapses [67]. A more efficient treatment would be frequent infusions of stem cell-derived beta cells, although this has achieved only limited success. iPSCs have been shown to be able to differentiate into beta cells in sufficient quantity to respond to changes in blood glucose levels [68]. Insulin genes have been successfully expressed in MSCs, but these cells have been unable to demonstrate the secretion of insulin [69]. It remains to be seen whether these therapies will satisfy the seven pillars of credibility for diabetes treatments [70]—including the cure of hypoglycaemia and the return of diabetes when transfused cells are removed. But as it stands, these criteria are still a long way off being met by stem cell-based therapies.

Clinical trials using stem cell therapies on human patients have so far produced mixed results. hESCs successfully differentiated into photoreceptors and retinal pigment epithelial cells were used to treat patients with dry age-related macular degeneration and Stargart’s macular dystrophy, resulting in a functional improvement in vision [71]. However, another trial using autologous adipose-derived stem cells to treat dry age-related macular degeneration in three patients reported severe vision loss [72]. The first clinical report of using hESC-derived cardiomyocytes to treat severe heart failure in a patient alongside a coronary artery bypass demonstrated an improvement in heart function, with a 10% improvement in ejection fraction (EF) [73]. Other attempts to treat heart failure with cardiomyocytes derived from adult tissue have shown promise in phase I trials, but raise specific concerns. For instance, c-kit-expressing cardiac progenitor cells found in the right atrium, once seen as a primary treatment method, have been shown to have low scalability in terms of cardiomyocyte production [74]. In one trial, a mean of 113 days was required to produce two million cardiomyocytes [75]. Traumatic spinal cord injury (SCI), for which treatment is currently limited to the administration of steroids and acute surgical interventions, can potentially be treated using adipose-derived MSCs. The capacity of MSCs to remyelinate, replace lost oligodendrocytes and rapidly replicate into progenitor cells, alongside their low immunogenicity, makes them ideal candidates to treat SCI [76]. A phase I trial using adipose-derived MSCs found no serious side effects from administration, and recorded an improvement in motor score in three out of eight patients [77]. Repeated intrathecal injections of MSCs, transplanted with bone marrow nucleated cells successfully improved motor function in an SCI patient who previously showed no improvement under standard therapy [78]. However, our understanding of the mechanism of action of MSCs and the pathology of SCI is still limited. A greater emphasis must now be placed on demonstrating the efficacy of phase I treatments via rigorously controlled, double blind, multi-centre clinical trials.

Even for existing treatments, such as using HSCT to treat SCD, there is still room for improvement in terms of donor compatibility and conditioning regimens used. SCD is a genetic disorder characterised by the distortion of haemoglobin-containing red blood cells into a rigid atypical crescent shape [79], with HSCT used as a means of restoring erythropoiesis. Use of HSCT to treat SCD is limited by the lack of allogenic donors, with less than 14% of SCD patients receiving a HSCT from a human leukocyte antigen (HLA)-matched sibling donor [80]. A HLA-matched sibling donor offers the best outcomes for SCD by improving engraftment and reducing the risk of post-transplant complications such as GvHD. This can be seen in the way in which increased HLA mismatching is correlated with higher mortality rates post-HSCT [81].

### The reporting of clinical trial and research outcomes

The pressure in stem cell science to demonstrate therapeutic value in clinical trials and stem cell research can manifest itself as an effort to spin their outcomes, for instance by focusing on statistically significant secondary outcomes to deflect away from adverse events or statistically insignificant primary outcomes [82]. According to the ISSCR, such reporting practices “distort medical and public interpretation of trial results” [3]. Whilst the expectation is that the findings of clinical trials and stem cell research will be published regardless of their significance, this is not always the reality.

Confusion surrounding the efficacy of clinical trials using autologous bone marrow stem cells to treat dilated cardiomyopathy prompted a group of researchers to investigate discrepancies from the reports in these clinical trials. The investigation found that clinical trial reports with the greatest number of discrepancies reported the greatest potential benefit to patients [83]. Using a definition for a discrepancy as conflicting statements regarding the study’s design, methods and results, the group identified 604 discrepancies in 133 reports from randomised controlled trials reporting on the effect of autologous bone marrow transplant on the EF for patients with dilated cardiomyopathy . EF is a measure of the volume of blood pumped out of the left ventricle during each contraction, and is assumed to correspond to heart function. In the five studies where the EF incremental increase was zero, there were no recorded discrepancies. This could point to a wider issue that clinical trial outcomes are misreported as the result of high therapeutic expectations.

Similar concerns were also aired around one of the most recent reported advancements in induced pluripotency, stimulus triggered acquisition of pluripotency (STAP) [84]. STAP was a remarkably simple model of applying stress stimuli such as low pH and physical pressure on cell membranes reported to induce pluripotency in mouse cells. After continual failures to reproduce STAP cells and doubts over the legitimacy of the technique, RIKEN conducted a misconduct investigation—discrediting the paper and finding its authors guilty of research misconduct. The STAP paper was subsequently retracted, with its authors concluding they were “unable to say without doubt whether the STAP-phenomenon is real” [85].

The STAP affair can be looked at from two perspectives. The first perspective is one which sees peer review as an effective tool in weeding out bogus papers. The second perspective is one which focuses on other falsified research reports which, unlike STAP, went undetected. Either way, misreporting on the levels as in the retracted STAP report can be seen as a symptom of the pressure on researchers to provide positive results in a field in which the appetite for innovation is magnified by inflated therapeutic expectations. The effect of such misreporting only serves to widen the gap between therapeutic expectation and reality.

## Conclusions

Although the politics, ethics and science of stem cell therapies have been treated as separate sources of regulation in stem cell science, their interrelationship is fundamentally important to understanding why stem cell therapies have failed to meet therapeutic expectations. The intensity of the ethical debate surrounding embryonic stem cell research has generated an unbridgeable impasse over the clinical role of hESC-based therapies. In seeking to circumvent the ethical debate, legislators have encouraged the development of more ethically justifiable therapies through tighter hESC regulation. However, anticipated stem cell therapies using somatic cells face significant translational hurdles in terms of the most basic safety and efficacy concerns, such as controlling the migration of transplanted cells, the compatibility of donors and the risk of tumorigenesis. In other words, stem cell ethics has directly influenced the selection of clinically unfit somatic-based therapies over embryonic-based therapies by shaping legislation.

Beyond the question as to whether placating our own moral convictions at the expense of the effectiveness of stem cell therapies is justifiable lies the danger that we are ignorant to the challenges facing novel stem cell-based therapies. Given the risks associated with ectopic stem cell treatments, our primitive understanding of both the mechanisms of such therapies and the pathologies of the diseases they treat, we should not be surprised by the therapeutic reality that all stem cell therapies beyond HSCT are experimental. Yet, the distortion of therapeutic expectations by unproven therapies and misreported clinical trial outcomes has created an impression that the application of stem cells in medicine is “limited”. In truth, the limitation of stem cells is the result of our own choice. By sidelining the cells with the greatest growth capacity and plasticity on the basis of moral objections, the regenerative potential of stem cells is left unfulfilled. It is this choice, above all, which has limited the application of stem cells in medicine.

### Additional files

Additional file 1: Department of Health Freedom of Information Request. (PDF 125 kb)

## Appendix 1

Political transparency in government funding is not only a crucial element of public policy, but aids the ethical discourse surrounding stem cell treatments by ensuring that research policy decisions which determine the direction of stem cell science are accessible. Released funding data (Table 1) promotes government accountability for policy decisions and evidences trends within the field of stem cell science through funding breakdowns.

**Table 1:** Estimates of funding for various research, condition and disease categories in the US

Research/disease areas	FY 2012 (actual)	FY 2013 (actual)	FY 2014 (actual)	FY 2015 (actual)	FY 2016 (estimated)	FY 2017 (estimated)
Stem cell research	$1374	$1273	$1391	$1429	$1495	$1495
Stem cell research, embryonic, human	$146	$146	$166	$180	$191	$191
Stem cell research, embryonic, non-human	$164	$154	$150	$159	$168	$168
Stem cell research, induced pluripotent stem cell, human	$175	$199	$280	$282	$296	$296
Stem cell research, induced pluripotent stem cell, non-human	$48	$43	$49	$61	$63	$63
Stem cell research, nonembryonic, human	$504	$431	$443	$445	$465	$465
Stem cell research, nonembryonic, non-human	$653	$613	$627	$632	$658	$658

Data provided by the National Institutes of Health (NIH), US (2016). Values are millions of US dollars and rounded. *FY* financial year

According to the Haldane Principle, individual funding decisions should be made by research councils rather than the government [86]. Whilst this allows for a degree of autonomy in the prioritisation of funding by researchers, this does not justify a complete absence of transparency in the block payments made by the Department of Health (DoH) to research councils including the National Institute for Health Research (NIHR). Through a Freedom of Information (FOI) request to the DoH for funding breakdowns over the past decade, it was revealed that the DoH “does not hold information on the department’s total expenditure on stem cell research, or a breakdown of such expenditure by area of stem cell science” [87]. Although research funding is allocated by the NIHR, funding for stem cell science is categorised under the wider funding bracket of “regenerative medicine”, meaning that no exact value for total government funding for stem cell science is known. A review of clinical trials using ATMPs authorised by the EU from 2004–2010 found that the majority were sponsored by either academia (50%) or charitable organisations (10%) [88]. A similar review in the UK would demonstrate the role of commercial sponsors in stem cell science, as well as the extent of pressure on academia to finance advances in the field. The absence of transparency around funding decisions may not directly impact the role of stem cells in medicine, but certainly limits the terms of the ethical debate waged around their use. How can we expect the merits and drawbacks of areas of stem cell science if the size of their role in regenerative medicine, and therefore their level of funding, is unknown? Without a baseline to measure changes in stem cell funding against and means to pinpoint an exact value of DoH funding for stem cell science, the direction in which funding decisions are taking stem cell science is far from clear.

## Appendix 2

### Neurological disorders

As one of the more effective treatments in animal models of ageing [89], nerve growth factor (NGF) has emerged as a potential treatment for Alzheimer’s disease (AD), a neurodegenerative disease characterised by the degeneration of cholinergic cells. NGF, unable to be transplanted into the brain by cannulation or pass the brain–blood barrier, could be carried by genetically modified cells able to diffuse across the brain–blood barrier. A clinical trial using genetically modified autologous fibroblasts showed no evidence of cognitive decline in 22 AD patients, with no adverse events reported after a 6-month follow-up [90]. Stem cells would be able to outperform adult fibroblasts in this function, given their higher migratory capacity [91] and mobility, meaning that NGF could be more distributed at a faster rate. Using NGF to treat AD represents a curative measure by maintaining cholinergic cell count, but further trials are warranted to ascertain the safety of this approach.

Multiple sclerosis (MS) is an autoimmune condition caused by the loss of oligodendrocytes (OLs), a myelin-producing glial cell. In terms of OL replacement strategies, some MS therapies have been successful in using neural progenitor cells (NPCs) to augment the re-myelenation capacity of the brain. A study using NPCs to treat a rat model of MS concluded that transplanted cells have a trophic effect increasing myelination by the host’s progenitor cells [92]. A cell line showing OL markers has also been derived using transduced NSCs [64].

NSCs may also be used in treating presymptomatic patients with Huntington’s disease (HD), an autosomal dominant disease for which much is still to be learnt regarding its pathogenesis. A study administering NSCs to rodents 1 week prior to and 12 h after a model of HD was imitated found that the prior transplantations demonstrated significant improvements in motor performance and limiting neuronal degeneration [93]. This technique, combined with advances in neuroimaging, could become an early intervention strategy for HD patients. However, clear risks remain and we need to ensure that the migration of NSCs to the pathological lesions caused by HD is safe, with the mechanism for the selective migration of NSCs still unclear [94]. Although the safety of this technique is yet to be defined, using stem cells to treat HD still remains one of the more effective therapeutic strategies.

### Toxicology

Outside of the traditional clinical research trajectory, stem cells represent a useful tool in developing in vitro cell lines to use for disease modelling and drug testing in order to anticipate toxicity in humans. Not only does this reduce dependency on animal models, but could aid our understanding of the pathogenesis of the diseases stem cell therapies are seeking to treat. For instance, iPSCs have been used to test for cardiotoxicity, hepatotoxicity and embryotoxicity [95]. Whilst we await the widespread availability of stem cell therapies, the toxicological application of stem cells serves as a useful reminder that stem cell technology already has a practical value in medicine.

## Abbreviations

AD: Alzheimer’s disease
AIFA: Agenzia Italiana del Farmarco
ATMP: Advanced therapy medicinal product
CAT: Committee for Advanced Therapies
DoH: Department of Health
EF: Ejection fraction
GvHD: Graft versus host disease
HD: Huntington’s disease
hESC: Human embryonic stem cell
HFEA: Human Fertilisation and Embryology Act
HLA: Human leukocyte antigen
HSCT: Hematopoietic stem cell transplantation
iPSC: Induced pluripotent stem cell
ISSCR: International Society for Stem Cell Research
MS: Multiple sclerosis
MSC: Mesenchymal stem cell
NGF: Nerve growth factor
NIHR: National Institute for Health Research
NSC: Neural stem cell
OL: Oligodendrocyte
SCD: Sickle cell disease
SCI: Spinal cord injury
SCNT: Somatic cell nuclear transfer
SHEEF: Synthetic human entity with embryo-like features
STAP: Stimulus triggered acquisition of pluripotency
