# Supplementary material for: The limited application of stem cells in medicine: a review
Source: Stem Cell Res Ther. 2018 Jan 2;9:1. doi: 10.1186/s13287-017-0735-7 (PMC5749007; doi:10.1186/s13287-017-0735-7)
Supplement: Supplementary file 1 — Department of Health Freedom of Information Request. (PDF 125 kb) [file 13287_2017_735_MOESM1_ESM.pdf]

Department of Health

09/11/2016

FOI-1058082

Dear Jordan Poulos,

Thank you for your request of 4th November 2016 under the Freedom of Information Act (2000). Your exact request was:

> *"I am currently writing an Extended Project Qualification titled "To what extent has the application of stem cells in modern day medicine been limited?". Due to the inaccessibility of government figures relating to the overall funding and funding breakdowns for stem cell science in the UK, I am making a freedom of information request to the department of health to release funding statistics for stem cell research over the last decade. This, if possible, should include the overall funding given by the Department of Health for stem cell research, but also the funding breakdowns in the various areas of stem cell science."*

I can confirm that the Department holds information relevant to your request.

The Department of Health does not hold information on the Department's total expenditure on stem cell research, or a breakdown of such expenditure by area of stem cell science.

The Department funds research through the National Institute for Health Research (NIHR) and the Department's Policy Research Programme (PRP). The PRP has not funded stem cell research during the past ten years.

Spend on research funded directly by the NIHR is categorised by Health Research Classification System (HRCS) health categories. There is no HRCS category for stem cell research, so we do not hold and would be unable to generate data on total NIHR spend on stem cell research, or a breakdown of such expenditure by area of stem cell science.

If you have any queries about this email, please contact me. Please remember to quote the reference number above in any future communications.

If you are dissatisfied with the handling of your request, you have the right to ask for an internal review. Internal review requests should be submitted within two months of the date of receipt of the response to your original letter and should be addressed to:

Head of the Freedom of Information Team
Department of Health
Room G18
Richmond House
79 Whitehall
London
SW1A 2NS

Email: freedomofinformation@dh.gsi.gov.uk

If you are not content with the outcome of your complaint, you may apply directly to the Information Commissioner (ICO) for a decision. Generally, the ICO cannot make a decision unless you have exhausted the complaints procedure provided by the Department. The ICO can be contacted at:

The Information Commissioner's Office
Wycliffe House
Water Lane
Wilmslow
Cheshire
SK9 5AF

Yours sincerely,

Alison Tingle

Freedom of Information Officer
Department of Health

freedomofinformation@dh.gsi.gov.uk
